# LIFETIME EXPOSURES AND COGNITION AT OLDER AGES: FINDINGS FROM THE WISCONSIN LONGITUDINAL STUDY

**DOI:** 10.1093/geroni/igae098.1354

**Published:** 2024-12-31

**Authors:** Michal Engelman, Gina Lee

**Affiliations:** Chair; Co-Chair

## Abstract

This symposium considers the relationship between later-life cognition and a range of social, economic, and environmental exposures throughout the life course. Drawing on over 65 years of rich population-based survey data and recent rigorous cognitive assessments from the Wisconsin Longitudinal Study (WLS), the papers in this symposium showcase empirical linkages between older adults’ cognitive health with a set of exposures including education, family socioeconomic status, occupational conditions, and familial connections. The first paper (Herd et al.) draws on the unique availability of adolescent IQ and high caliber cognitive diagnostic data that distinguishes between underlying etiologies to examine the relationship between early life IQ, educational attainment, and later life dementia. The paper by Topping links the wealth of WLS data on early life socioeconomic background and high school experiences to patterns of change in later life cognition, with attention to both cognitive change and mechanisms of selection. Next, Qin and Engelman draw on the WLS’s sibling data to consider the relationship between later-life cognitive health and educational differentials among siblings, and Lee et al. consider the impact of sibling relationships on later life cognition. Finally, Williams et al. draw on the WLS survey, genetic, and cognitive data to consider the interaction between ApoE-4 status, employment-based environmental exposures in midlife, and later life pollution in shaping dementia risk. Taken together, the papers in this symposium showcase the enormous potential of combining longitudinal survey data with cognitive assessment for learning about the life course determinants of later-life cognitive health.

